# 

**Published:** 1814-12

**Authors:** 


### MONTHLY CATALOGUE OF MEDICAL BOOKS.

MEDICO-CHIRURGICAL Transactions, published by the Medical and Chirurgical Society of London. Volume V. 8vo.; plates; 18s.

Pathological Researches. Essay I. on Malformations of the Human Heart; illustrated by numerous Cases, and five Plates, containing fourteen Figures; and preceded by some Observations on the Method of improving the Diagnostic Part of Medicine. By J. R. Farre, M.D. 8vo. 7s.

A System of Operative Surgery, founded on the Basis of Anatomy. By Charles Bell, Esq. Surgeon to the Middlesex Hospital, &c. &c. The second Edition; 2 vols. 8vo. 1l. 18s.

A Dissertation on Gun-Shot Wounds. By Charles Bell, Esq. Surgeon to the Middlesex Hospital, &c. &c. 8vo. 10s. 6d.

Observations on the principal Diseases of the Rectum and Anus, particularly Structure of the Rectum, the Hæmorrhoidal Excrescence, and Fistula in Ano. By Thomas Copeland, Esq. Second Edition, considerably enlarged. 8vo. 7s.

Appendix to J. Callow's Modern Catalogue, containing the new Works published during the present Year; to which is added, a large Collection of Foreign Books lately imported, to be sold at reduced prices.

*Books imported by J. Callow.*

Alibert Description des Maladies de la Peace, observées à l'Hôpital Saint-Louis; et Exposition des mailleures méthodes suivies pour leur traitement grand in fol. pap. vél fig. color. 9 livraisons.

Belloc Cours de Médecine Légale, Theorique et Pratiqué, 8vo.

Mémoires de la Société Médicale d'Emulation. 7 tom. in 8vo. fig. 1798-1811.

Ploucquet, Litteratura, Medica Digesta, sive Repertorium Medicinæ Practicæ, Chirurgiæ, atque rei Obstetriciæ. 4 tom.

Scarpa Traité Pratique des Hernies, ou Memoires Anatomiques et Chirurgicaux sur les Maladies, traduits de l'Italien, par M. Cayol, avec planches, en folio.

Hippocrates Aphorismi et Prænotionum liber Græce et Latiné edante par Bosquillon. Paris, 1814. 18mo.

---

### TO CORRESPONDENTS.

*We have found it necessary to curtail several Communications received this Month. We take the opportunity of suggesting, that brevity is* one *of the perfections of composition, and should always be kept in view where the sense of the writer is not likely to be obscured by it.*

*The Letter of Chirurgicus savours too much of quackery to be inserted. We strongly suspect its real signature should have been* EMPIRICUS OCULARICUS, *and the term* friend to science *might have admitted a corresponding change.*

*The answer to Dr. Sutton has been again postponed on account of the the length of the various Communications this Month. Our first duty is to discharge the obligations we are under to our numerous friends, by as early an insertion of their papers as possible.*

*Communications have reached us from Dr. Mitchell, Messrs. Smerdon, Walker, R. B. H., &c. &c.*

### INDEX

### TO THE THIRTY-SECOND VOLUME.

ABERNETHY, Mr. J. on Mr. Hunter's theory of life 419
Adams, Dr. Jos. on the hereditary properties of diseases 140
————, on insects as the cause of itch 497
————, on scrofula 499
Adam, Mr. J. on the effects of a vegetable poison 364
Adams, Sir W. strictures on his treatment of cataract 486
Ægineta, account of 303
Ætius, historical notice of 302
ANALYSIS, CRITICAL, of new publications 47, 140, 227, 322, 403, 507
Animals, experiments on 58
Animation, suspended, efficacy of oxygen gas in restoring 36
Apothecaries, their report of diseases 82, 170
Armstrong, Dr. J. on puerperal fever 507
Arsenic, white oxide of, soluble in water 253
————, effects of, on the stomach 428
Ascites, case of 26
Ashford, Mr. on the influence of air in shut sacs 208
Assalini, Mr. on the effects of ligatures on arteries 445
Axbridge, Mr. on Mr. Jones's case of inflammation of the abdominal viscera 89
Ayre, Dr. Jos. on venesection in diabetes 128
Babad, Dr. success of, in curing intermittents by bleeding 345
Balfour, Dr. W. on the reunion of separated parts 411
Batchelder, Dr. J. his case of disease of the heart 307
Baths, account of an establishment of, at Bristol 61
Beclard, M. on the fœtus respiring the liquor amnii 521
Belladonna, cases of poisoning with 390
Benamor, Dr. death of, recorded 257
Bernard, Mr. J. his case of compression of the brain 8
————, on the effects of vaccination in hooping cough 256
Bertrand, M. his experiments with charcoal 258
Bethlem Hospital, remarks on the bad management of 124
Bile, extravasation of, into the cavity of the abdomen 152
Bill for regulating mad-houses, observation on the 2
Blood, analysis of 222
Books in the press, and preparing for publication 170, 257, 526
—— published 88, 176, 264, 352, 528
Brain, portion of, lost, recovery after 185
——, protruded, case of 525
Brenan, Dr. on puerperal fever 403
Brodie, Mr. C. on animal heat 307
Brown, Dr. W. C. on the condition of navy surgeons 416
Burroughs, Mr. G. F. on inflammatory fever 267
Calcagno, Dr. on the internal use of charcoal 428
Calculi, biliary, remedy for 169
Caloric, extrication of, during the coagulation of the blood 224
Cancer, remedy for 110, 255, 524
Carcinoma recti, symptoms of 246
Carmichael, Mr. on the venereal disease 229
CASES—of premature parturition 100
———— hydrothorax 101, 134
———— ascites 26
———— eruption 102
———— hysteria 104
———— jaundice 106
———— abscess of the liver 119, 370
———— vomica 120
———— diabetes 129
———— premature puberty 145
———— contracted wrist 146
———— cynanche laryngea 147
———— bile extravasated into the abdomen 152

Cases—of trismus 31, 164
——— spontaneous combustion 164
——— laryngitis 166
——— compression of the brain 8
——— fungus hæmatodes 19
——— ulcerated larynx 25
——— pyrosis 27
——— acute rheumatism 28
——— a foreign body found within the heart 37
——— retention of urine 43, 520
——— injury of the brain 184
——— phlegmasia dolens 187
——— ruptured uterus 194
——— hydrophobia 217, 431
——— tic douloureux 219, 433
——— rheumatism of the heart 220
——— chancre 232
——— polypus uteri 266
——— inflammatory fever 269
——— over dose of opium 272
——— diseases of the heart 305, 307, 389, 448, 514
——— intermittent fever 345
——— poison 364, 390
——— tænia 395
——— pemphigus 397
——— puerperal fever 404, 462
——— reunion of separated parts 410
——— affection of the stomach from arsenic 428
——— diabetes 446
——— syphilis 449
——— extra uterine pregnancy 459
——— gout 491, 493
——— hemiplegia 525
——— protrusion of the brain ib.
——— spina bifida 526
Cataract, observations upon 65
Celsus, his medical and surgical precepts commended 117
Chancres, case of 232
Channing, Dr. W. on puerperal fever 461
Charcoal, medicinal effects of 248, 428
Chatelain, M. his analysis of a concretion in the jejunum 80
Chausier, M. on extra-uterine pregnancy 459
Clarke, Mr. C. M. on diseases of females attended by discharges 161, 239, 336
Clayton, Mr. on a convulsive epidemic 32
Clivers, efficacy of, in the cure of cancer 255
Colchicum autumnale, beneficial effects of, in gout and rheumatism 78, 312, 393, 491
Cold applied to the abdomen, beneficial in inflammation of the viscera, note, 90
Collectanea Medica 36, 128, 217, 314, 395, 496
Colville, Mr. E. his case of tic douloureux 219
Combustion, spontaneous, case of 164
Consumption, on the nature and treatment of 227
Cooper, Mr. A. P. his case of premature puberty 145
Cornish, Mr. on a convulsive epidemic 32
Croup, treatise upon, by M. Double 47
Cupping, suggestions respecting 476
Cynanche laryngea, case of 147
Delametherie, M. on the progress of the sciences in France 132, 222
Deaths in the bills of mortality 82, 170
Death, mode in which it is occasioned by hanging 469
Diabetes relieved by emetics 446
Diarrhœa, from an ulcerated state of the intestines 374
Dickson, Dr. on pemphigus 397
Digestion, facts concerning 257
Diseases in London, report of 82, 170, 353, 441
——— Paris 255
Dislocations, remarks on 473
Double, M. his treatise on croup 47
Drugs, London prices of 83, 171
Earle, Mr. on a disease affecting the meatus urinarius in females 427
———, his remedy for retention of urine 520
Eau medicinale, efficacy of, in gout 493
Emetics, use of, in diabetes mellitus 446
Ergot, or secale cornutum, natural history of 90
———, its effects in uterine cases 94, 190
Farre, Dr. J. R. on malformations of the human heart 513
Farr, Dr. S. on the elements of medical jurisprudence 327
Females, on the diseases of, attended with discharges 161, 239, 336
Fever, yellow, proofs of its being contagious 45
———, inflammatory, symptoms of 267
———, intermittent, cured by bleeding 345
———, puerperal, cured by oil of turpentine 404
———, observations on 461, 507
Feminine disease attacking men described 166
Flesh, means of preserving, in gases 435
Fœtus in the abdomen of a boy, history of a 81
———, its capability of breathing the liquor amnii 521

Fothergill, Dr. S. on scarlatina anginosa 481
Fractures, new mode of treating 171
———, observations upon 470
Fradin, M. his apparatus for obviating the effects of breathing impure air 81
Friend's Retreat, excellent regulations of 127
Fryer, Mr. his case of bile extravasated into the abdomen 152
Fungus hæmatodes of the kidnies, case of 19
———, poisonous effects of 364
Galen, historical notice of 300
Gallois, Dr. le, on the vital principle 56
Galloway, middle granite district of, described 75
Gaultier, M. his account of the poisonous effects of belladonna 390
Gillespie, Dr. L. on an indigenous remedy for cancer 109
Gilpin, Dr. on the contagious nature of yellow fever 45
Gordon, Dr. J. on the extraction of caloric during the coagulation of the blood 223
Gorham, Dr. J. on a disease of the heart 305
———, on diarrhœa 375
Gout, efficacy of colchicum in the cure of 312, 393, 491
———, on purgatives in 381
———, effects of eau medicinale on 493
Graduates in medicine at Glasgow, list of 258
Guersent, M. on the poisonous effects of belladonna 390
Guinea pigs, peculiarity in their natural history 61
Hæmorrhage, uterine, restrained by ergot 97
———, effects of opium upon 153
Hale, Dr. E. on the production of animal heat by respiration 233
Harrison, Dr. on an amended bill for regulating mad-houses 1
———, elected physician to the Northern Dispensary 257
Harrup, Mr. his case of wounded brain 185
Hepar sulphuris, good effects of, in tettery eruptions 165
Heart diseases, and dissections of 39, 221, 305, 307, 389, 448, 513
Heat, animal, observations upon 233, 295
Hemiplegia, case of 525
Hippocrates, his pre-eminence in medicine and surgery 114
Hildebrant, M. on the preservation of heat in gases 435
Hodgson, Mr. his case of contracted wrist 146
Holcus cafer, a plant resembling the sugar cane, described 346
Home, Sir Everard, on the influence of the nerves upon arterial pulsation 248
Hosack, Dr. D. on the surgery of the ancients 111, 297
Hugo, Mr. on small-pox after vaccination 478
Hydrothorax, case of 101, 134
Hysteria, case of 104
Insects, observations on the eyes of 315, 400, 500
Itch, remedy for 79
———, observations on insects as a cause of 497
Intelligence, Medical and Philosophical 74, 162, 248, 343, 427, 520
Jaundice, case of 106
Jaymes, M. his case of vomica 120
John, Dr. on the analysis of vegetable substances 434
Kentish, Dr. E. on his establishment of baths, &c. at Bristol 61
Kerrison, Mr. his case of hydrophobia 431
——— tic douloureux 433
Kinglake, Dr. on vaccination 180
Klaproth, Mr. on the solubility of white oxide of arsenic in water 253
Labour-pains excited by ergot 95
Laryngitis, case of 166
Larynx, ulcerated, case of 25
Lectures announced.—On Medicine—Dr. Adams, Dr. Babington, Dr. Cholmeley, Dr. Curry, and Dr. Freer 258
——— Dr. Clutterbuck and Dr. Pearson 259
——— Anatomy, Surgery, and Physiology—Dr. Spurzheim 82
——— Mr. Cline, Mr. Cooper, Dr. Jeffrey, Mr. Taunton 258
——— Mr. Brookes, Mr. Shute 259
——— Mr. Brodie, Mr. Carpue 347
——— Mr. Pettigrew 348
——— Midwifery and Diseases of Women and Children—Dr. Haighton, Mr. Towers, Mr. Want 258
——— Dr. and Mr. Clarke, Dr. Clough, Dr. Merriman 259
——— Dr. Squire 170, 347
——— Dr. Ramsbotham 259, 348
——— Dr. Davis 348, 526

LECTURES announced.—On Chemistry, Botany, and Materia Medica—Mr. Allen, Dr. Babington, Dr. Brown, Dr. Cleghorn, Dr. Cholmeley, Dr. Curry, Dr. Marcet 258
———— Dr. J. Davy, Dr. R. Harrison, Dr. Roget 259
———— On the Teeth—Mr. Fox 258
———— On the Eye and Ear—Mr. Stevenson 436
Leucorrhœa, observations on 239
Liver, abscess of 119, 371
Locked jaw, efficacy of Ol. Terebinth. in 345
Lumbrici, remarks upon 347
Lunatics, on the treatment and condition of 123
————, on the act relative to 4
Madeira House, at Bristol, establishment of a 62
Magendie, M. experiments of 343
Maingault, Dr. on vomiting 517
Marquais, M. on vomiting 518
Marshall, Mr. John, on premature parturition by puncturing the membranes 100
————, Mr. H. on hydrophobia 217
Mathias, Mr. A. elected consulting surgeon to the Northern Dispensary 257
Meadow saffron, effects of, on cattle 216
Measles, instance of, occurring twice in one individual 256
Medical reform, observations upon 323
———— jurisprudence, elements of 327
Medico-chirurgical Transactions, vol. iv. critical analysis of 65, 145
Melville, Mr. account of his diving machine 522
Membranes, puncture of, useful in certain cases 101
Meteorological register 87, 175, 263, 351, 439, 527
Monthly catalogue of medical books 88, 176, 264, 352, 440, 528
Montegre, M. on digestion 257
————, on lumbrici 347
Moore, Dr. F. on phlegmasia dolens 187
Moxa, application of, beneficial for incontinence of urine 346
Nauche, M. on diseases of the bladder 155
Nerves, effects of their division on various functions 58
————, influence of, on the beating of arteries 248
Neville, Mr. his case of the bad effects of arsenic on the stomach 428
Notices to correspondents and readers 176, 264, 352, 440, 528
Opium, use of, in uterine hæmorrhage 153
————, in an over-dose of, bad consequences of bleeding in 271
Opium, use of, in gangrene attacking the toes 430
Oxygen gas, efficacy of, in restoring suspended animation 36
Parturition, premature, utility of puncturing the membranes in certain cases of 100
Pears, Dr. C. on pulmonary consumption 227
Pemphigus occurring epidemically 165
————, observations upon 281, 397
Penkivil, Mr. J. on rheumatism of the heart 220
Percival, Dr. E. on cynanche laryngea 147
Peripneumonia, observations upon 358
Peritonitis, puerperal, relieved by cold applications, note, 90
Pharmacopœia, London, remarks upon 10
Phlegmatia dolens, case of 187
————, observations upon 524
Physic-nut, medicinal properties of 110
Plates 99, 177, 265
Pollock, Dr. W. on tænia 395
Polypus uteri, new instrument for the removal of 265
Pradier, M. account of his remedy for gout 523
Prescot, Oliver, A.M. on the secale cornutum or ergot 90
Pregnancy, extra uterine, case of 459
Prices of drugs 84, 170
———— of substances used in pharmacy 84, 172, 260, 348, 437
Prize medals, proposed 76, 521
Puberty, premature, case of 145
Purgatives, efficacy of, in scarlatina anginosa 483
Pyrola umbellata, effects of 163
Pyrosis, case of 27
Pyrophorus, on the formation of 436
Rabbits, facts relating to their natural history 61
Ramsbotham, Dr. J. on sudden death soon after parturition 135
————, on rupture of the uterus 194
Rance, Mr. T. F. on fungus hæmatodes of the kidnies 19
Remedy for cancer 111
Report of the progress of the sciences in France 132
Respiration, influence of temperature upon 132
Reynard, M. his case of abscess of the liver 119
Rheumatism, acute, case of 28
————, remedy for 163
————, cured by oil of turpentine 520
Rigby, Mr. on vaccinating with one puncture 107

Robertson, Mr. A. his case of hydrothorax 134
——, Dr. W. on peripneumony 358
Rousselot's powder, composition of, and effects in cancerous ulcers 524
Sankey, Mr. on phlegmasia dolens ib.
Savery, M. on pemphigus 281
Santer, Mr. his new mode of treating fractures 177
——, his improved instrument for removing polypus uteri 265
Sciences, progress of, in France 132, 222
Scrofula, observations upon 499
Scarlatina anginosa, observations upon 483
Secale cornutum, history and effects of 91, 94
Semple, Mr. on a disease of the liver 371
Serres, Mr. de, on the eyes of insects 314, 400, 500
Small-pox, cases of, after vaccination 478
Smerdon, Mr. on Sir W. Adams's treatment of cataract 487
Society, Royal, proceedings of 74, 162, 248
——, Linnæan 75
——, Wernerian ib.
——, Royal Medical of Edinburgh 76
——, Kirwanian, of Dublin 77
——, Westminster Medical 427, 520
——, Medical, of London 520
Southcott, Joanna, remarks on her case 311
Spurzheim, Dr. his lectures on the brain 82
Spina bifida, fatal case of 526
Stewart, Mr. on opium in uterine hæmorrhage 153
Stomach passive in vomiting 430
Surgery, historical facts relating to 112, 297
Surgeons in the navy and army, remarks upon the condition of 416
Sutton, Dr. on the eau medicinale 195
——, on purgatives in gout 381
Sweden, state of vaccination in 250
Syphilis, on diseases confounded with 229, 449
Taunton, Mr. J. his case of retention of urine cured by puncturing the bladder 43
Terebinthinæ oleum, efficacy of in locked jaw 345
—— in puerperal fever 404
—— in acute rheumatism 520
Thomson, Dr. on a substance extracted from the vagina 249
Tic douloureux, remedies for 219, 433
Tænia, cured by decoction of pomegranate 395
Tongue, enlarged, partial extirpation of 169
Tonsils, enlarged, improved method of removing 475
Travers, Mr. B. on cataract 65
Trismus cured by bleeding 31, 164
Upas, on the poison of 343
Urine, retention of, cases of 43, 346, 520
Uterus, effects of ergot upon the 94
——, some affections of, described 240
Vaccination, causes of failure in 180
——, observations upon 107, 213, 289
Vaginæ procidentia, symptoms of 243
Vagina, account of a substance extracted from the 249
Varicose veins in the urethra and bladder 155
Vesicæ procidentia, account of 243
Venesection, effects of, in diabetes 129
Vital principle, experiments and observations upon 57
Vomica, case of, cured by the operation for empyema 121
Vomiting a symptom of cancer of the uterus 240
—— excited by injecting emetic tartar in the veins 425
——, observations upon 517, 519
Walker, Mr. R. on the London Pharmacopœia 10
——, on vaccination 211, 289
——, on specific remedies 293
——, on fractures 470
——, on dislocations 473
——, on removing tonsils 474
——, on cupping 476
——, Dr. J. R. on emetics in diabetes 446
Want, Mr. on a remedy for gout 78, 201, 312, 393, 491
——, on the effects of meadow saffron on cattle 216
——, his monthly report of diseases 353, 441
Wardley, Mr. his case of hydrothorax 101
Witter, Mr. S. on the effects of oxygen on suspended animation 36
Wigard, Dr. his remedy for croup 80
Wood, Mr. W. his case of a foreign substance in the heart 37
Woodham, Mr. on the discrepancy of medical opinions 183
——, on hypotheses 457
Wrist, contracted case of 146
Yeatman, Mr. on the effects of bleeding in an over-dose of opium 271

### PLATES.

Secale Cornutum or Ergot - - - - - Page 99
Fractures of the Extremities - - - - - - 177
Improved Instrument for the Removal of Polypus Uteri - 265

